# Influence of Rural Non-Smoking Adolescents’ Sense of Coherence and Exposure to Household Smoking on Their Commitment to a Smoke-Free Lifestyle

**DOI:** 10.3390/ijerph10062427

**Published:** 2013-06-13

**Authors:** Olalekan A. Ayo-Yusuf, Masego M. Rantao

**Affiliations:** Department of Community Dentistry, School of Dentistry, Oral & Dental Hospital, Faculty of Health Sciences, University of Pretoria, Pretoria 0001, South Africa; E-Mail: masego.rantao@up.ac.za

**Keywords:** smoke-free households, adolescents, sense of coherence, South Africa

## Abstract

This 18-month longitudinal study examined the influence of adolescents’ sense of coherence (SOC) and exposure to household smoking on their commitment to a smoke-free lifestyle. This study investigated a representative sample of 8th graders from 21 randomly selected high schools in the rural Limpopo Province of South Africa (n = 2,119). Of the total sample of 2,119 participants, 294 (14%) reported smoking at baseline and were therefore excluded from further analysis. Of those who did not smoke at baseline, 98.1% (n = 1,767) reported no intention of smoking in the upcoming 12 months. Of those who completed follow-up and had no intention of smoking at baseline (n = 1,316), 89.1% still did not smoke and remained committed to being smoke-free. Having a lower SOC, reporting alcohol binge-drinking at baseline, and having a household member who regularly smokes indoors (OR = 0.46: 0.26–0.82), as compared to not having any smoker in the household, were associated with lower odds of honoring a commitment to a smoke-free lifestyle. Furthermore, those who identified themselves as black Africans, as opposed to belonging to other race groups, were more likely to maintain a smoke-free lifestyle. Our findings suggest that interventions to prevent adolescent smoking should prioritize stress-coping skills and promote smoke-free homes.

## 1. Introduction

Half of all life-time smokers, most of whom reside in low- and middle-income countries, will die prematurely due to smoking, losing 20 to 25 years of their life expectancy [1]. In addition, smokers face a greater risk of developing various cancers than non-smokers do [2,3,4]. In South Africa, cigarette smoking already accounts for about 45,000 or 8–9% of deaths that occur annually [3]. In addition, smoking is ranked third out of 17 mortality risk factors evaluated in the country [5]. Given that most adult smokers began smoking as adolescents, it is important to explore ways to prevent the initiation of tobacco use among youths. Adolescence is a particularly critical period for tobacco use prevention, because efforts to prevent or delay smoking initiation can be effective during this life phase [6]. Considering these facts on tobacco-related mortality and morbidity [1,2,3,4,5], the current past-month smoking prevalence of 21% among South African adolescents remains unacceptably high [7]. Nevertheless, the smoking prevalence of 17.9% among adolescent black Africans, who constitute the majority of the South African population, is still relatively low when compared to the smoking prevalence of 34.4% among adolescent whites or 35.9% among adolescent Coloureds (those of mixed ancestry) during 2008 [7]. The black African population, particularly those in the rural areas whose smoking rates are even lower, could therefore be seen as a future market by the tobacco industry. As a result, on-going tobacco control efforts remain a high priority for promoting health in rural South Africa. 

Most of the studies that have been previously undertaken to understand the predictors of cigarette smoking among adolescents have focused on adolescents in high-income countries [8]. Nonetheless, most of these predictors have also been found to be common for adolescents in lower income countries [9,10]. Predictors of smoking include the smoking status of peers, siblings and parents, family structure, low socio-economic status of parents, low refusal self-efficacy, low self-esteem, depression vulnerability, lack of participation in religious and sporting activities, engagement in other risk behaviors and a pro-smoking environment [8,9,10,11,12,13]. On the one hand, some adolescents have been reported to smoke cigarettes as a response to coping with stress and depression [8,13]. On the other hand, stress has been found to be more common among adolescents from households with low socio-economic status [8], which in turn has been associated with a higher likelihood of having a smoker in the household [14]. Therefore, theoretically, the propensity to cope with stress may protect against smoking initiation in low-income communities.

Several studies have been conducted to predict tobacco use among adolescents, but relatively few studies have been done on why adolescents choose to remain smoke-free. Current predictors of adolescents who choose to remain smoke-free include reduced exposure to smoking in the household, having a parent with a higher education status and having health-related concerns regarding cigarette smoking [15,16,17]. Household smoking rules have also been identified as a deterrent to adolescent smoking which can keep youths smoke-free well into their adult years [17,18]. Smoke-free homes in particular also reduce exposure to second-hand smoke and associated risks. Other variables found to be significant predictors of the intention not to smoke included fewer friends who smoke, and perceived behavioral control to remain smoke-free [18]. The few studies conducted in South Africa on predicting tobacco use among adolescents have been cross-sectional studies and have focused mainly on adolescent smoking and its predictors [9,10], rather than on understanding the vast majority of adolescents who elect not to smoke cigarettes. Previously published longitudinal studies on predictors of adolescents who plan to remain smoke-free have also been limited to resource-rich settings with historically high smoking prevalence and/or have not been theoretically driven [13,14,15,18]. 

Several theories view smoking initiation as both intentional (e.g., theory of planned behavior) [19] or as automatic/reactive (prototype/willingness model) [20]. However, the theory of planned behavior (TPB) has been the main theory used to explain smoking behavior among adolescents [19,21]. According to this theory, adolescents’ behaviors are determined by their intentions and their perceived control over their actions. This perceived behavioral control (self-efficacy) is a perception that a behavior is within one’s own control. Although a cross-sectional study in South Africa, noted ethnic differences in the extent to which self-efficacy influences smoking [10], it nevertheless stands to reason that while some adolescents may intentionally choose to smoke cigarettes, other adolescents may intentionally choose not to smoke, and take control of maintaining such a decision through a display of strong cigarette-offer refusal self-efficacy. This study hypothesizes that adolescents with strong self-efficacy or strong perceived behavioral control to resist smoking are more likely to stay committed to a smoke-free lifestyle than those with a weaker self-efficacy. As an extension to the perceived behavioral control, this study also looks at the theory of salutogenesis [22]. The concept of salutogenesis applies focus on how individuals use resources at their disposal to stay healthy. The key to this theory is to harness the factors that maintain health, as opposed to factors associated with health risks, by applying problem-solving skills and using available resources [22]. The main construct in the salutogenic theory is sense of coherence (SOC). SOC unlike other similar constructs such as resilience or locus of control can be applied across different cultures to measure individuals’ personal way of living and capacity to respond to stress [22,23]. The SOC construct posits that stress is inherent to human nature, but that people’s global orientation to coping with stress determines whether they remain healthy or not [22]. A previous study indeed demonstrated that SOC moderates the influence of school-related stress among adolescents [24]. This study therefore also hypothesizes that adolescents who have a higher propensity to cope with stress, or who have a higher SOC, would be more likely to maintain an intention to staying smoke-free because they would be able to better identify generalized resistance resources [23] to help them to cope effectively with life stressors without resorting to negative adaptive behavior such as smoking. 

Hence, considering that SOC has also been suggested as a universal self-efficacy [25], this longitudinal study sought to determine the association between non-smoking adolescents’ sense of coherence and their commitment to remaining smoke-free, independent of the presence of a smoker in the adolescents’ household. Findings from this study may contribute to a better understanding of the behavior among non-smoking adolescents and may contribute towards the selection of appropriate priority interventions to prevent adolescent smoking in the thus far understudied rural population which is the focus of the current study. 

## 2. Methods

### 2.1. Study Design and Study Population

This study involved a provincially representative sample of 8th graders (in the first year of high school and they are average age 14 years (13–15 years), but in rural areas, they tend to be a little older because children are only sometimes allowed to attend school when they are able to do the long walk to school) from public secondary schools in the largely rural Limpopo Province of South Africa. The participants in this longitudinal study were recruited from 8th graders in all the 21 randomly selected secondary schools (n = 2,119; 87.9% response rate). The students were originally involved in a tobacco use prevention trial. The details of the sampling procedure have been published previously [26]. Briefly, schools were selected using a two-stage cluster sampling design, with 33 school districts as the sampling frame, from which one school was randomly selected per district. The data for this analysis were collected from a three-wave survey. Structured questionnaires were administered, first at baseline (April 2005), then at six months (September 2005) and then 12 months later (September 2006). This analysis was restricted to those adolescents who reported that they were non-smokers in the first wave, and reported an intention not to smoke in the 12 months following the survey (n = 1,767). The same participants completed the questionnaire at baseline, 6 months, and 18 months after baseline. Participants who were classified as followed up were those who participated in all three survey waves of the study. 

### 2.2. Measures and Definitions

The same self-administered questionnaire was used to obtain the same information from the same participants in each wave, such as each respondent’s socio-demographic profile, vulnerability to depression, alcohol and dagga use, tobacco use status, household member tobacco use status and a participant’s intention to smoke in the coming 12 months and propensity to cope with stress, as measured on a SOC scale. 

#### 2.2.1. Socio-Demographic Characteristics 

Each study participant provided information about his or her age, gender, ethnicity (black African or other), and family living structure (for example, whether a respondent lived with either parent or both parents in the same household or not). A participant’s socio-economic status was measured by the mother’s reported highest level of schooling attended, which was categorized as either less than 12 years, 12 years, or more than 12 years of schooling. In addition, similar to what was done in a previously published study [27], participants were asked about the type of house they lived in (whether they lived in formal or informal housing). 

#### 2.2.2. Depression Vulnerability

This study also measured vulnerability to depression among the study participants in the recent past. The depression screening item used was similar to one previously demonstrated as a valid proxy measure of depression vulnerability [28]. Briefly, participants were asked: “During the past 12 months, did you ever feel so sad or hopeless almost every day for two weeks or more in a row that you stopped doing some usual activities?” Those who answered in the affirmative were classified as being depression-vulnerable. Similar a previously published study [29], because this study did not use a direct measure for stress, depression vulnerability was used to infer the impact of stress as experienced by adolescents. 

#### 2.2.3. Sense of Coherence

Respondents’ sense of coherence (SOC), in other words, their propensity to cope with stress, was measured using a six-item adapted Antonovsky SOC scale [22] previously validated for this population [30], with a satisfactory reliability (Cronbach’s alpha = 0.68). Sample item question: “How often do you have feelings that you’re not sure you can keep under control?” Each of the items were scored on a seven-point continuum using a likert-type scale with response options anchored with “never” (coded 7) on one end, and “very often” (coded 1) on the other end. Because the variable was used as a continuous measure, a high score represented a stronger SOC. Considering that SOC is not fully stable in adolescence [22], similar to the approach used in a previous study [30], change in SOC (SOC development) was computed by subtracting the baseline SOC scores from that of the follow-up SOC scores. 

#### 2.2.4. Tobacco and Other Substance Use

Using items previously used in national and international youth risk behavior surveys [7,26], the respondents were categorized as current smokers and snuff users if they indicated having smoked a cigarette or used snuff respectively in the 30 days preceding the date of the survey. Alcohol binge-drinking was assessed by asking how often during the 30 days preceding the survey the participants had five or more drinks of alcohol in a row [7]. Marijuana (*dagga*) use was assessed based on a similar question on the use of *dagga* at least once in the last 30 days [7]. 

#### 2.2.5. Socio-Environmental Influences for Tobacco Use

As reported in a previously published paper [26], peer influences related to tobacco were assessed, including friends’ use of cigarettes. Family influences considered included household members’ smoking status. Those who reported household member smoking had to indicate whether the household members smoke mostly indoors or mostly outdoors [26]. The potential influence of the school environment was assessed by asking: “Is there a school rule against tobacco in your school?” The response options provided were “There is no official/written rule”, “There is a rule, but it is not enforced”, “There is a rule, but it is only sometimes enforced” or “There is a rule and it is strictly enforced”. Participants were asked whether they had been taught during the current school year that “smoking makes your teeth yellow, causes skin to wrinkle or makes you smell bad?” Furthermore, participants were asked about the frequency of participation in sporting and religious activities [7,26]. 

#### 2.2.6. Future Smoking Intention and Self-Efficacy to Refuse Cigarette Offers

Participants’ intention to smoke cigarettes was assessed by asking “At any time in the next 12 months, do you think you will try to smoke a cigarette?” The response options were “Definitely not”, “Probably not”, “Probably would” and “Definitely would”. Those who responded “Probably would” and “Definitely would” were classified as “Not committed to a smoke-free lifestyle”. Those who responded “Definitely not” and “Probably not” were classified as “Committed to a smoke-free lifestyle”. 

To assess adolescents’ perceived behavioral control (self-efficacy), participants were asked how confident they were in refusing a friend’s offer of a cigarette. The question used was similar to that employed in past published studies [24,31,32]: “If one of your best friends were to offer you a cigarette, would you smoke it?” The four response options were “Definitely not” (coded 4), “Probably not” (coded 3), “Probably would” (coded 2), and “Definitely would” (coded 1). This factor was measured on a continuous scale. The higher the respondent’s score, the higher the respondent’s cigarette offer refusal self-efficacy.

The main outcome measure “Commitment to a smoke-free lifestyle” was defined as having reported the intention not to smoke at baseline, still remaining abstinent at both 6 months and 18 months after baseline, and also reporting the intention not to smoke in the following year.

### 2.3. Data Analysis

Given the cluster sampling method employed in this study, statistical analyses were performed using the “svy” command option in the chosen statistical package, STATA Version 10 (Stata Corp, TX, USA). The “svy” command allows statistical commands to be executed that take into account the survey design, such as the cluster sampling method used in the study. For the bivariate analysis, cross-tabulations were used to determine associations between potential categorical predictor variables at baseline and the outcome variable of interest. Group differences were tested using chi-square statistics. T-tests were then used to test for associations between the continuous variables and the outcome variable of interest. Statistical tests were two-tailed, and the threshold for statistical significance was set at *p* < 0.05. All the variables that were associated with a commitment to a smoke-free lifestyle at *p* ≤ 0.05 were included in the multiple logistic regression analysis. Odds ratios (OR) with a 95% confidence interval were used in estimating the effect sizes. All the participants with missing data on the outcome variable were excluded from the final analysis. SOC development was included in the multivariate analysis, while controlling for baseline SOC. An adjustment for baseline SOC was made to control for potential floor effects that may constrain declines in the scores of participants with a low baseline SOC score, and ceiling effects that may constrain increases in the scores of participants with a high baseline SOC score [30]. Furthermore, because some of the study participants were exposed to a school-based curriculum tobacco use prevention program, assignment to the control or intervention groups was also included as an independent variable to adjust for any potential effect of the program, irrespective of the level of statistical significance. 

## 3. Results

The sample used in this study consisted mostly of black African adolescents (89.6%). Of the total sample, 50.1% were female. The mean age of the study participants at the baseline of this study was 14.6 years (range 12 to 19 years; 75.9% were ≤ 15 years). Of the total sample of 2,119 participants, 294 (14%) reported smoking at baseline and were therefore excluded from the analysis, as indicated above. 

**Figure 1:**
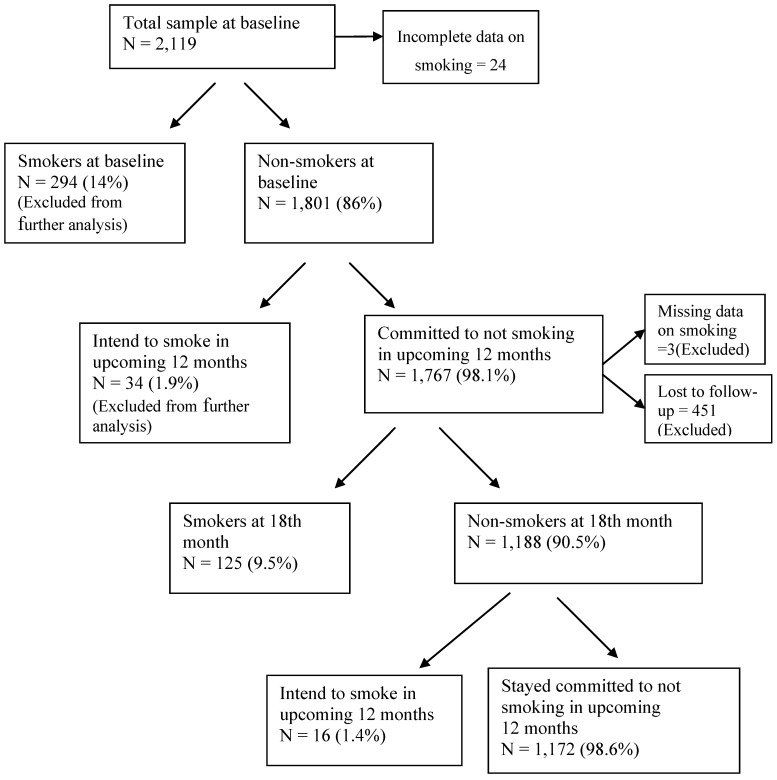
Classification of study participants into smokers, non-smokers, those intending to smoke and those not intending to smoke.

Attrition analysis showed that the only significant difference between the baseline characteristics of those followed-up on and the participants lost to follow-up was that those lost to follow-up were significantly older. The main reason for lost to follow-up was leaving current school to move to another school in a different region and a few were reported to have stopped schooling to seek employment mostly on farms. Figure 1 presents an outline of the classification of the study participants according to their smoking status and intention to smoke in the upcoming 12 months. At baseline, 86% (n = 1,801) of the adolescents did not smoke cigarettes. Of those who did not smoke at baseline, 98.1% (n = 1,767) had no intention of smoking in the upcoming 12 months. Of the 1,316 adolescents available for the follow-up, 90.5% still did not smoke, and 97.9% of these non-smokers (that is, 89.1% of those baseline non-smokers on whom a follow-up was done) remained committed to remaining smoke-free. 

There was no significant age difference between those adolescents who were committed to a smoke-free lifestyle and those who did not make such a commitment (14.5 *vs*. 14.6 years; *p* = 0.40). Similarly, there was no significant difference in baseline cigarette-offer refusal self-efficacy between those adolescents who were committed to a smoke-free lifestyle and those who did not indicate such a commitment. Table 1 represents the bivariate analysis of adolescents who were committed to a smoke-free lifestyle and honored that commitment. Adolescents who did not report binge-drinking and those who did not have a household member who smoked at baseline were more likely to have honored their commitment to not smoking than those who drank alcohol or those who had family members that smoked. Those with a higher mean SOC development were significantly more likely to stay committed to a smoke-free lifestyle than those with a lower mean SOC development (*p* = 0.04).

**Table 1:** Prevalence of those who remained committed to a smoke-free lifestyle by baseline characteristics of the participating adolescents.

Baseline characteristics (n)	% Baseline non-smokers remaining smoke-free	*p*-value
Control group (657)	88.8	
Intervention group (656)	89.7	
		0.66
Male (625)	87.5	
Female (688)	90.8	
		0.11
Black African (1,204)	89.6	
Other race (101)	83.2	
		0.01
Mother < 12 years schooling (567)	88.7	
Mother = 12 years schooling (310)	91.3	
Mother > 12 years schooling (439)	88.4	
		0.40
Informal housing (158)	89.9	
Formal housing (1,152)	89.2	
		0.78
Lives with neither parent (353)	88.7	
Lives with one parent (508)	90.6	
Lives with both parents (447)	87.9	
		0.40
No regular sport participation (925)	90.1	
Participates in sport regularly (380)	87.1	
		0.12
Doesn’t regularly attend religious ceremonies (667)	87.9	
Regularly attends religious ceremonies (628)	90.5	
		0.14
No marijuana use in past month (1,295)	89.4	
Marijuana use (16)	75.0	
		0.06
No snuff use (1,212)	89.5	
Snuff use (75)	84.0	
		0.14
No alcohol binge-drinking (1,231)	90.3	
Alcohol binge-drinker (82)	73.2	
		<0.01
Not depression-vulnerable (1,075)	89.8	
Depression-vulnerable (233)	86.3	
		0.12
Most friends do not smoke (1,277)	89.2	
Most friends smoke (24)	87.5	
		0.79
No household member smokes (823)	91.5	
Household members smoke outdoors (320)	86.9	
Household members smoke indoors (120)	81.3	0.01
No school rule against smoking (516)	88.6	
Rule, but not enforced (380)	89.2	
Rule sometimes enforced (147)	90.5	
Rule strictly enforced (261)	89.7	
		0.92
Taught about effects of smoking at school (671)	89.4	
Not taught about effects of smoking (465)	89.9	
Not sure (164)	87.2	
		0.62

In a multivariable-adjusted model (Table 2), the factors that remained positively associated with commitment to a smoke-free lifestyle were being a black African and having a stronger SOC at baseline and a higher SOC development over the study period. Adolescents were less likely to commit to a smoke-free lifestyle if at baseline they were binge-drinking and had a household member who regularly smoked indoors than if there was no smoker in the household. Those who had a smoker in the household, but reported that the smoker usually does not smoke indoors, tended to be less likely to commit successfully to a smoke-free lifestyle. However, the difference between the latter group and those without a smoker in the household was not statistically significant.

**Table 2:** Final logistic regression model of factors independently associated with adolescents’ commitment to a smoke-free lifestyle.

Characteristics	OR	95% CI	*p*-value
Control	1		
Intervention	1.14	0.73–1.78	0.56
Black African	1		
Other race group	0.58	0.37–0.90	0.02
No alcohol binge-drinking	1		
Alcohol binge-drinking	0.33	0.19–0.58	<0.01
No household member smokes	1		
Household member smokes outside	0.68	0.41–1.13	0.13
Household member smokes inside	0.46	0.26–0.82	0.01
Baseline mean SOC (per unit increase)	1.28	1.05–1.56	0.02
Mean SOC development (per unit increase)	1.04	1.01–1.07	0.01

## 4. Discussion

This study used a representative sample of rural South African adolescents to test two hypotheses: (1) that adolescents with strong self-efficacy or strong perceived behavioral control to resist smoking are more likely to stay committed to a smoke-free lifestyle than those with a weaker self-efficacy; and (2) that adolescents who are able to cope with stress, or who have a higher SOC, are more likely to remain smoke-free, independent of if there is a smoker in the adolescents’ household. This study demonstrated that one in ten of the adolescents who initially intended not to smoke cigarettes later reported that they had taken up smoking. In relation to the first hypothesis, self-efficacy was not found to be a significant predictor of commitment to a smoke-free lifestyle. This finding is in contrast to previous studies’ findings [19,21]. The difference between the current findings and those of prior studies may be related to the fact that, unlike with past study cohorts, the outcome of this study was on staying smoke-free. In addition, this study controlled for adolescents’ SOC, which has been considered a source of universal self-efficacy [25]. Furthermore, the adolescents in the current study were essentially non-smokers at baseline and had the intention of not smoking. They are therefore conceivably at relatively low risk of taking up smoking. Also, previous studies among black African adolescents suggest that cigarette refusal self-efficacy may not be as significant a predictor of smoking in this population as in other population groups in South Africa [10] or elsewhere, because of the relatively low levels of smoking in this population group in general [32]. It is pertinent to note that the relatively low prevalence of smoking in this study cohort is also reflected in the fact that very few reported having many friends who smoke. It was therefore not surprising that peer smoking did not have a significant influence on staying smoke-free. The finding that adolescent alcohol binge-drinkers in this study were also less likely to stay committed to a smoke-free lifestyle is consistent with a recent study’s finding that early drunkenness is a risk factor for adolescent smoking [33]. 

In relation to the second hypothesis, this study showed that a strong ability to cope with stress or a strong SOC appears to protect these rural adolescents against smoking initiation. Consistent with the literature [23,34,35], this study’s findings show that adolescents with a higher SOC were more likely to commit to a smoke-free lifestyle than those with a lower SOC. Evidence does indeed suggest that confidence about having the means to cope with stress may be related to behavioural intentions [22]. Thus adolescents who may experience stress and have a negative predisposition to coping would indeed be more susceptible to smoking than adolescents who may have a positive predisposition to coping, as measured by a stronger SOC. Conversely, adolescents who are able to cope better and would be more resilient to life’s pressures may be more likely to use their personal and environmental resources to maintain their health [23,34]. Considering that irrespective of the adolescents’ SOC levels, those living in a smoking home environment were less likely to stay smoke-free, also provide further evidence of the importance of the household social contexts of a non-smoking environment as a protective factor. Like some previous studies [13,16,36], this study’s findings suggest that adolescents whose family members smoke cigarettes are more likely to become smokers than adolescents with predominantly non-smoking family members. This suggests a need to encourage and provide conditions under which homes can be made smoke-free in order to protect adolescents and young children from taking up smoking. Evidence in developed countries suggests that adults who support smoke-free homes are more likely to be people who also support smoking bans in public places [37]. Smoke-free homes can be enabled by applying stricter rules against smoking in the home or specifically prohibiting smoking indoors [17]. 

Having stricter rules against smoking or against smoking indoors could help to prevent adolescent uptake or an escalation of smoking. Unlike in a previous study where complete restrictions on household tobacco use were tested [38], the current study shows that partial restrictions, such as permitting smoking outside the house only, may reduce the influence of household members’ smoking on tobacco use among adolescents, but may not be enough to eliminate that influence. Household smoking rules, apart from reducing children’s exposure to toxins in smoke, also have the potential to create a form of anti-tobacco socialization and thus build an environment supportive of non-smoking. Conceivably, such a non-smoking family environment may also be conducive to the development of adolescents SOC [39]. 

In interpreting the above findings, it is necessary to keep in mind a few limitations. One limitation of the current study is that the data applies mainly to rural adolescents who were present in school at the time of data collection. The findings may therefore not necessarily extend to out-of-school adolescents or to more urban settings. Another limitation is that a single item measure was used to measure the intention to smoke and refusal self-efficacy, whereas some other studies have use multiple-item measures. Some single item measures using the theory of planned behavior have, however, provided good predictions of intentions [18,21]. Furthermore, considering that only baseline characteristics were used as covariates, it is possible that the 18 months interval between when the baseline measures were collected and when the final outcome measure was obtained might have attenuated the association between these variables. Another limitation is that the findings from this study are based on self-reports by students about their own tobacco use behavior. This may lead to under- or over-reporting of their habits. In addition, social norms may have discouraged the students from writing about their personal beliefs. However, we do not expect this to change the conclusions reached in this study significantly, as it has been shown that self-reports of tobacco use are generally accurate, especially if the questionnaires are self-administered [40].

## 5. Conclusions

Despite the limitations of the study, the findings of this study support the hypothesis that higher SOC is significantly associated with rural adolescents’ commitment to staying smoke-free, independent of the exposure to smoking home environment. However, this study failed to demonstrate a significant influence of cigarette-offer refusal self-efficacy as operationalized in this study. Taken together, the findings suggest that the SOC construct was theoretically better than self-efficacy construct in explaining rural adolescents’ commitment to staying smoke-free. This study’s findings also highlight the need to extend interventions for the prevention of adolescent smoking initiation to include promoting smoke-free home environments. Finally, given the influence of SOC on smoking susceptibility, life-skills training that include coping skills would be a useful intervention to help encourage rural South African adolescents to stay smoke-free. 
